# Manipulable Objects Facilitate Cross-Modal Integration in Peripersonal Space

**DOI:** 10.1371/journal.pone.0024641

**Published:** 2011-09-19

**Authors:** Michiel van Elk, Olaf Blanke

**Affiliations:** Laboratory of Cognitive Neuroscience, Brain Mind Institute, École Polytechnique Fédérale de Lausanne, Lausanne, Switzerland; Royal Holloway, University of London, United Kingdom

## Abstract

Previous studies have shown that tool use often modifies one's peripersonal space – i.e. the space directly surrounding our body. Given our profound experience with manipulable objects (e.g. a toothbrush, a comb or a teapot) in the present study we hypothesized that the observation of pictures representing manipulable objects would result in a remapping of peripersonal space as well. Subjects were required to report the location of vibrotactile stimuli delivered to the right hand, while ignoring visual distractors superimposed on pictures representing everyday objects. Pictures could represent objects that were of high manipulability (e.g. a cell phone), medium manipulability (e.g. a soap dispenser) and low manipulability (e.g. a computer screen). In the first experiment, when subjects attended to the action associated with the objects, a strong cross-modal congruency effect (CCE) was observed for pictures representing medium and high manipulability objects, reflected in faster reaction times if the vibrotactile stimulus and the visual distractor were in the same location, whereas no CCE was observed for low manipulability objects. This finding was replicated in a second experiment in which subjects attended to the visual properties of the objects. These findings suggest that the observation of manipulable objects facilitates cross-modal integration in peripersonal space.

## Introduction

From morning to night we use many objects that extend our bodily capabilities and that make our life much easier. We use a knife to butter our bread, make notes with a pen, prepare dinner using cooking utensils and brush our teeth with a toothbrush. In some cases these objects can even be considered as an extension of the human body [1], for instance when tennis players report ‘viewing the racket as an extension of their arm’ or in the case of upper limb amputees who can attain an amazing degree of control over neural prostheses and who often consider the prosthesis as a part of their own body [2].

In recent years several studies have elucidated the neural mechanisms supporting multisensory integration during tool use in more detail. For instance, by using single-cell recordings in monkeys it was found that the response properties of visuo-tactile neurons in the anterior intraparietal sulcus (aIPS) changed after the monkey acquired the skill to use a tool as a rake [3]. Whereas the initial receptive field of these neurons responded selectively to visual stimuli presented near the hand, after training with the tool the receptive field of these neurons was found extended into more distant space surrounding the end of the tool. In humans comparable effects of tool use have been established, by investigating the interference effect of distractor lights presented near the end of the tool on the discrimination of tactile stimuli applied to the hand [4], [5], [6], [7], [8]. In this task subjects respond faster when the spatial position of the distractor light is congruent compared to incongruent with the felt vibration (i.e. up or down), which is known as the cross-modal congruency effect (CCE). The cross-modal congruency effect is considered a measure of multisensory processing in peripersonal space, i.e. the space directly surrounding one's body. Thus the finding that the cross-modal congruency effect extends towards the end of the tool suggests that tool use indeed extends one's peripersonal space [4].

Most studies on tool use and peripersonal space have typically used novel tools with which the subject had only little experience. However, as indicated above, in daily life we use many objects with which we have profound experience and that extend our bodily capabilities as well. The last decade many studies have shown that conceptual knowledge about familiar objects is strongly associated to motor representations specifying the use of the objects. For instance, at a behavioral level it has been found that the mere presentation of pictures or words referring to graspable objects results in the priming of the handgrip that is appropriate for grasping the object [9], [10], [11]. Furthermore, neuroimaging studies have shown that the retrieval of conceptual knowledge about the use of objects is accompanied by activation in premotor and parietal brain areas, that are associated with actually using the objects [12], [13], [14], [15], [16].

Besides these affordance-based effects, several studies have shown that familiar objects can facilitate the allocation of spatial attention. For instance, it was found that the presentation of task-irrelevant pictures of manipulable objects resulted in a facilitated detection of targets presented at the same location as the object [17]. In addition, it has been observed that functional object information (i.e. which object needs to be grasped first?) can be automatically inferred when two objects are correctly positioned for action [18], [19], [20]. For instance, patients with spatial neglect could report the ‘active’ item of an object pair that was spatially arranged for action (e.g. a corkscrew near the top of a wine bottle), thereby overriding their spatial bias to the ipsilesional side [20]. By using a temporal order judgment task with healthy participants it was similarly found that active objects were perceived earlier when the objects were positioned for action [21]. On the basis of these findings it has been suggested that functional information about the use of objects is processed pre-attentively, thereby resulting in a visual processing advantage for the active target of object pairs.

In sum, the studies discussed thus far show (1) that using novel tools extends one's peripersonal space and supports the integration of multisensory information and (2) that viewing manipulable objects activates relevant spatial and motor representations, supporting the actual use of these objects. Given these findings, an intriguing question is whether manipulable objects facilitate the integration of multisensory information in peripersonal space as well. That is, when using everyday objects, like a hammer or a pair of scissors, these objects often feel as an extension of our body and peripersonal space may be extended or projected towards these objects [4]. Because the CCE is enhanced when visual distractors are presented near the hands or near the tips of tools [4], [22], the CCE is considered a reliable measure of multisensory processing in peripersonal space. Accordingly, it could well be that the CCE is enhanced as well when visual distractors are presented near pictures representing familiar manipulable objects.

To test this hypothesis, in the present study subjects were presented with pictures representing objects that differed in their manipulability. Some objects could be easily manipulated and were highly associated to specific hand actions (*i.e. high manipulability*; e.g. a toothbrush, a mug or a cell phone), some objects could be easily manipulated but are not used as frequently (*i.e. medium manipulability*; e.g. car keys, a soap dispenser, tweezers) and some objects were more difficult to manipulate and are typically not associated to a specific action (*i.e. low manipulability*; e.g. a computer screen, a chalkboard, a candle holder). The object pictures were presented on a screen and visual distractors were superimposed on the pictures. The vibrotactile stimulation was applied to the subject's right hand and subjects responded by indicating the location of the felt touch with their left hand (for a similar CCE-setup, see: [23]). If manipulable objects facilitate cross-modal integration in peripersonal space, a stronger CCE is expected (i.e. a stronger difference between congruent and incongruent visual distractors) for objects that can be easily manipulated compared to objects that are more difficult to manipulate. In the first experiment subjects were explicitly required to retrieve action semantic information about the object pictures, by answering a question about the action associated with the object after each picture. In the second experiment, subjects were required to attend to the visual properties of the object pictures, by answering a question about what the object looked like. In this way it was investigated whether the observation of manipulable objects automatically modulates multisensory integration, or whether it requires the retrieval of action semantic information (for a similar manipulation, see: [24]).

## Materials and Methods

### Experiment 1 Materials and methods

#### Participants

In the first experiment 16 subjects participated (4 females, mean age = 20.8 years), who received 10 CHF for participation. Subjects declared themselves through informal verbal inquiry to be right-handed. Both experiments were approved by the local ethics committee: La Commission d'ethique de la recherche Clinique de la Faculté de Biologie et de Médecine – at the University of Lausanne, Switzerland. All subjects verbally gave informed consent prior to participation and were fully debriefed after the experiment. Owing to the non-invasive, purely behavioral nature of our study, the ethics committee considered verbal consent was appropriate and approved this consent procedure. The study was conducted in accordance with the declaration of Helsinki.

#### Stimuli

As stimuli we selected pictures from the Bank of Standardized Stimuli (BOSS; see: [25]). This database contains 480 standardized color pictures of everyday objects that are rated for familiarity, visual complexity and manipulability. For the present study we selected 120 pictures of objects (see Appendix S1). Based on the manipulability ratings these objects were split into three different categories: low manipulability (e.g. computer screen), medium manipulability (e.g. soap dispenser), and high manipulability (e.g. cell phone). Independent t-tests confirmed that the three categories did not differ in familiarity (p's>.50) or visual complexity (p's>.20), but the categories differed in manipulability ratings (p's<.001).

#### Design and procedure

**Figure 1:**
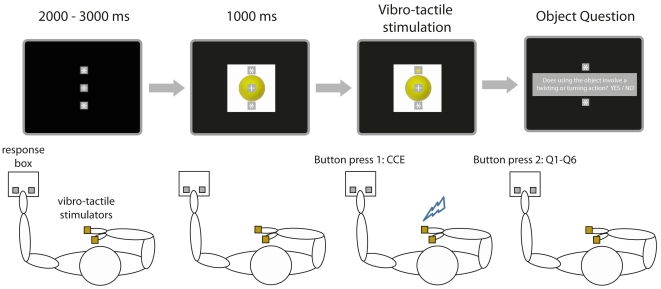
Experimental setup and procedure. Subjects were seated behind a table, facing a computer screen. Tactile vibrators were attached to the thumb and index finger of the subject's right hand and the subject responded with the left hand by pressing one of two buttons on a button box. Each trial started with a fixation cross (1^st^ panel from left), followed by the presentation of an object picture (e.g. a tennis ball; 2^nd^ panel from left), visual distractor and tactile stimulation (3^rd^ panel from left) and an object question (right panel). Subjects responded by indicating whether the thumb or index finger was stimulated (Button press 1) and by answering a question about the object (Button press 2).

A schematic overview of the experimental setup and procedure is represented in Figure 1. Subjects were seated behind a table facing a computer screen. Custom made vibrotactile stimulators were attached to the thumb and index finger of the subject's right hand. Subjects were instructed to place their right hand on the table during the experiment and to hold the index finger above the thumb at a distance of approximately 5 cm. A serial response box was placed on the left side of the table to measure the subject's responses.

During the experiment a white fixation cross and two asterisks, presented 70 pixels above and below the fixation cross, were continuously visible on the screen. At the beginning of each trial the fixation cross and the asterisks were presented for 2000–3000 ms against a black background. Next a picture representing an object appeared in the background. Object pictures were centrally presented at a resolution of 400×400 pixels. Subjects were required to identify the object so that they could answer a question about the action associated with the object (see below).

1000 ms after the onset of the picture a visual distractor was presented for 100 ms (i.e. one of the asterisks turning yellow) followed by a vibrotactile stimulation for 100 ms (i.e. there was a temporal delay of 100 ms between the visual distractor and the tactile stimulation). Importantly, the vibrotactile stimulation could be *congruent* with the visual distractor (e.g. visual distractor presented above the fixation cross and vibrotactile stimulation applied to the index finger) or *incongruent* with the visual distractor (e.g. visual distractor presented above the fixation cross and vibrotactile stimulation applied to the thumb). Each object picture was presented twice, once with a congruent visuo-tactile stimulation and once with an incongruent visuo-tactile stimulation. In total the experiment consisted of 240 trials plus an additional 16 practice trials at the beginning of the experiment. Subjects were required to indicate whether the tactile stimulation was applied to their thumb or index finger by pressing the left or the right button of the response box respectively with their left hand. After the subject responded the picture disappeared from the screen and a question appeared. If the subject did not respond to the vibrotactile stimulation within 3000 ms the picture was removed from the screen and a question appeared.

For the first experiment we used 6 different questions about the action associated with the object (see Table 1) that were pseudo randomly presented. The same question was never presented more than twice in a row. The mapping of yes/no responses to the left or right button was counterbalanced across the different questions. The question remained on the screen until the subject made a response, upon which the next trial was initiated.

**Table 1:** Object questions used in the different experiments.

*Experiment 1: Action Questions*
Q1: Does using the object involve a pushing action?
Q2: Does using the object involve a twisting or turning action?
Q3: Does using the object involve a lifting action?
Q4: Does using the object involve a back-and-forth action?
Q5: Does using the object involve a squeezing or pinching action?
Q6: Does using the object involve a movement towards your body?
*Experiment 2: Visual Questions*
Q1: Does the object contain plastic parts?
Q2: Does the object contain metal parts?
Q3: Does the object have a round shape?
Q4: Does the object have a square shape?
Q5: Is the object colored?
Q6: Is the surface of the object smooth?

In the first experiment subjects answered questions about the action associated with the object (upper part). In the second experiment subjects answered questions about the visual properties of the object (lower part).

For the analysis, trials with incorrect responses and trials that exceeded the subject's mean by more than two standard deviations were excluded from analysis. To control for speed accuracy trade-offs reaction times and error rates were combined in one measure, the inverse efficiency (IE), by dividing the reaction times by the proportion of correct trials per condition [26], [27]. Data were analyzed using a repeated measures ANOVA with the factors congruency (Congruent vs. Incongruent trials) and Object type (low, medium and high manipulability objects). Analysis focused on differences in the cross-modal congruency effect (CCE; i.e. the difference between incongruent and congruent trials) between the different stimulus categories (i.e. objects with low, medium and high manipulability respectively), which should become apparent in an interaction between Congruency and Object type.

### Experiment 2 Materials and Methods

#### Subjects

In the second experiment 15 right-handed subjects participated (3 females, mean age = 21.2 years) who received a financial remuneration for participation.

#### Experiment 2 Methods

The experimental design was the same as in Experiment 1. However, instead of answering questions about the action associated with the object, participants were required to answer a question about the visual properties of the object (see Table 1).

## Results

### Experiment 1 Results

#### Cross-modal congruency task

Behavioral data from the first experiment is represented on the left side of Figure 1. Errors and missed responses occurred in less than 1% of all trials. The analysis of the inverse efficiency (IE) during the cross-modal congruency task revealed a main effect of congruency, F(1,15) = 13.2, p<.005, η^2^ = .47, reflecting faster responses for congruent (876 ms, SE = 90 ms) compared to incongruent trials (922 ms, SE = 86 ms) and thereby confirming that the basic congruency manipulation was successful. Importantly, a significant interaction was observed between congruency and object type, F(2,30) = 4.0, p<.05, η^2^ = .21, reflecting that the CCE differed between different stimulus categories. Post-hoc t-tests revealed no significant CCE for objects with low manipulability ratings (p>.66), whereas a significant CCE was observed for objects with medium manipulability, t(15) = −4.2, t<.001, and for objects with high manipulability, t(15) = −2.4, p<.05.

#### Object Questions

Analysis of the reaction times to the object questions revealed that subjects tended to respond slower to questions about objects with medium manipulability (2004 ms) and high manipulability (1986 ms) than to objects with low manipulability (1873 ms; t = −2.1, p = .051 and t = −1.8, p = .10 respectively).

In addition, we used the subjects' responses to the action questions to cross-validate the manipulability ratings of the pictures that were collected in a previous study [25]. To this end we calculated per object and action question the ratio between ‘yes’ and ‘no’ responses as follows: (nr. of yes-responses – nr. of no-responses)/(nr. of yes–responses+nr. of no-responses). The ratios were averaged per object across the different questions. In this way we obtained a normalized *action index* per object: low scores reflect that only few action features applied to the object, high scores reflect that many action features applied to the object. A highly significant correlation was observed between the manipulability ratings provided by the BOSS and the action index obtained in the present experiment, Pearon's r = 0.71, p<.001. This finding suggests that the previous ratings can be cross-validated in a different country, with a different pool of subjects and a different methodology. Most importantly, this finding suggests that the assignment of objects to different categories based on the manipulability ratings is warranted.

#### Control for object size

Finally we were interested in the question whether the difference in the cross-modal congruency effect could partly be attributed to visual differences between the stimuli used in the experiment. Although the different stimulus categories did not differ in visual complexity, by definition objects that can be easily manipulated with one's hand (e.g. a hairbrush) are smaller in size than objects that are more difficult to manipulate (e.g. a computer screen). For each picture we calculated the object size in terms of the total number of pixels (i.e. the number of pixels excluding the white background). The number of pixels provides a rough estimation of the actual object size. As expected, it was found that objects with high manipulability were smaller in size (average number of pixels = 29716, SD = 1460) than objects with medium manipulability (average number of pixels = 36348, SD = 1794) and objects with low manipulability (average number of pixels = 49520, SD = 1570).

To control for the possible confound that the difference in the CCE between stimulus categories could be partly attributed to differences in stimulus size, we conducted an additional analysis. Pictures in each stimulus category were classified as representing small objects or large objects, based on a median split on the object size. An ANOVA was performed on the inverse efficiency data with the factors congruency (Congruent vs. Incongruent trials), Object type (low, medium and high manipulability objects) and Object Size (small, large). Importantly, object size did not interact with congruency (F<1), suggesting that the CCE was not modulated by object size.

### Experiment 1 Discussion

In the first experiment a stronger crossmodal congruency effect (CCE) was observed for pictures representing medium and high manipulability objects compared to objects that were not strongly associated to an action. Typically, the CCE is taken to reflect the ease of integrating multisensory information in peripersonal space [4], [5], [7], [28]. The present findings suggest that seeing pictures of manipulable objects facilitates the multisensory processing of stimuli presented near the hand and the object. This finding extends previous studies that have shown a stronger CCE when visual distractors are presented at the distal part of a tool [3], [4], [5], [6], [7]. In addition, the finding of a CCE for manipulable objects extends previous studies that have shown that observation of these objects activates the relevant motor representations required for actually grasping the object [9], [10], [14], [24]. The present study indicates that besides priming low-level motor features, manipulable objects facilitate the integration of multisensory information in peripersonal space as well.

In the first experiment each object picture was followed by an action question about the action associated with using the object. Thus, when viewing the object picture, subjects may have been engaged in a process of motor imagery, thinking about how they would actually use the object. As a consequence, it is unclear if the stronger CCE for manipulable objects is primarily related to this motor imagery process (e.g. the subject imagines interacting with the object, thereby facilitating the processing of information in peripersonal space; cf. [29], [30]) or whether crossmodal integration is automatically modulated by simply viewing a picture of the object. To investigate this question, in a second experiment we used the same experimental setup as in the first experiment, but instead of asking a question about how to use the object, subjects answered a question about what the object looked like (e.g. ‘is the object colored?’).

### Experiment 2 Results

#### Cross-modal congruency task

**Figure 2:**
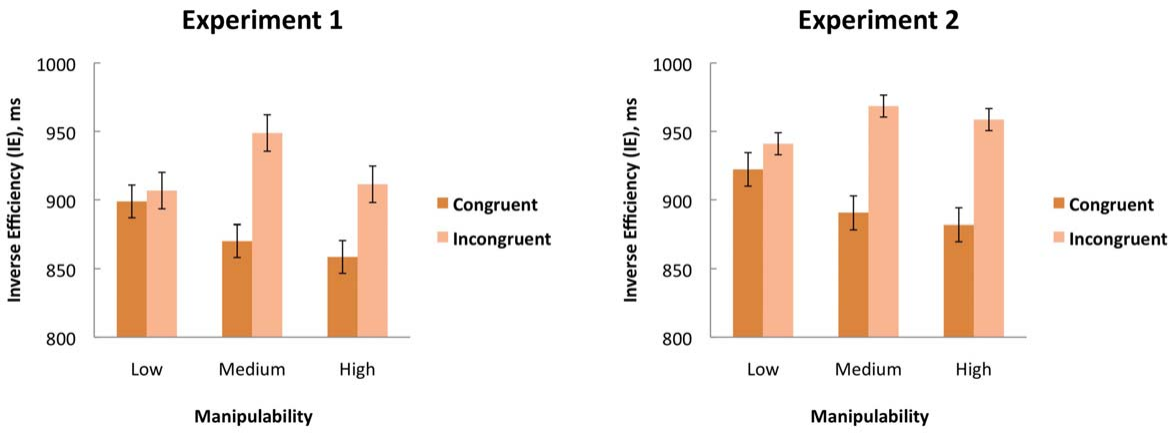
Behavioral data of Experiment 1 and 2. Inverse efficiency data of Experiment 1 (left panel; attend to action features) and Experiment 2 (right panel; attend to visual feature) for the cross-modal congruency task to pictures representing objects with low (left bars), medium (middle bars) and high manipulability (right bars). Dark bars represent responses to congruent visuo-tactile stimulation and bright bars represent responses to incongruent visuo-tactile stimulation. Error bars represent standard errors.

Behavioral data from the second experiment is represented on the right side of Figure 2. Errors and missed responses occurred in less than 1% of all trials. The analysis of the inverse efficiency (IE) during the cross-modal congruency task revealed a main effect of congruency, F(1,14) = 14.4, p<.005, η^2^ = .51, reflecting better responses for congruent (898 ms, SE = 121 ms) compared to incongruent trials (956 ms, SE = 116 ms). Similar as in the first experiment, a significant interaction was observed between congruency and object type, F(2,28) = 4.4, p<.05, η^2^ = .24, reflecting that the CCE differed between different stimulus categories. Post-hoc t-tests revealed a significant CCE for objects of medium manipulability, t(14) = −3.6, t<.005, and for objects of high manipulability, t(14) = −6.5, p<.001, whereas no significant CCE was observed for objects with low manipulability ratings (p>.46).

#### Object Questions

Analysis of the reaction times to the object questions revealed no significant differences between responses to objects of low manipulability (1127 ms), of medium manipulability (1094 ms) and of high manipulability (1110 ms; p's>.26).

#### Control for object size

To control for the possible confound that the difference in the cross-modal congruency effect was related to differences in the size of the object represented in the picture, we again conducted an additional analysis. For each category the pictures were classified according to the object size in terms of absolute number of pixels. An ANOVA was performed on the inverse efficiency data with the factors congruency (Congruent vs. Incongruent trials), Object type (low, medium and high manipulability objects) and Object Size (small, large). Importantly, object size did never interact with congruency (F<1), suggesting that the CCE was not modulated by object size.

#### Between-experiment comparison

To compare the findings between Experiment 1 and 2 an additional ANOVA was conducted on the CCE data with congruency (Congruent vs. Incongruent trials) and Object type (low, medium and high manipulability objects) as within-subject factors and Experiment (1 vs. 2) as a between-subjects factor. No interaction was found between Experiment and any of the other factors and no main effect of Experiment was found, suggesting that the CCE modulation by object manipulability and the overall reaction times were comparable between Experiment 1 and 2.

In addition, we compared the reaction time data to the object questions between Experiment 1 and 2, using Object type (low, medium and high manipulability objects) as within-subjects factor and Experiment (1 vs. 2) as a between-subjects factor. First, a main effect of Experiment, F(1, 29) = 16.8, p<.001, reflected faster responses to the object questions in Experiment 2 (1110 ms, SE = 148) compared to Experiment 1 (1954 ms, SE = 143). In addition, an interaction between Experiment and Object Type, F(2, 58) = 3.2, p<.05, reflected that whereas for Experiment 1 responses to different Object Types differed (i.e. faster responses to low manipulability compared to medium and high manipulable objects), in Experiment 2 responses to different Object Types were comparable.

### Experiment 2 Discussion

In the second experiment it was investigated to what extent the observation of manipulable objects automatically results in facilitated cross-modal integration. Rather than asking subjects questions about the action associated with the object, in the second experiment subjects answered a question about the visual properties of the object. Similar to the first experiment a stronger cross-modal congruency effect was observed when subjects observed pictures representing objects of medium or high manipulability compared to objects of low manipulability. This finding suggests that the observation of manipulable objects automatically facilitates cross-modal integration and thereby extends previous studies showing that object pictures activate relevant motor programs for grasping [9], [10], [14], [24].

## Discussion

Following the notion that one's peripersonal space can be extended or projected towards tools, in the present study we investigated whether the observation of pictures representing everyday manipulable objects would result in facilitated cross-modal integration. A stronger cross-modal congruency effect was found for pictures representing objects that could be easily manipulated (e.g. a toothbrush) compared to objects that were more difficult to manipulate (e.g. a computer screen). This effect was observed both when subjects were explicitly required to retrieve the action information associated with the object (Experiment 1) and when subjects were only required to attend to other action-unrelated properties of the object (Experiment 2). These findings suggest that the mere observation of manipulable objects facilitates the integration of cross-modal integration in peripersonal space.

Previous studies have shown that the observation of manipulable objects results in the automatic retrieval of action information required for actually interacting with the object. For instance, at a behavioral level it has been found that the observation of pictures or words referring to manipulable objects primes the hand grips associated with grasping the object [9], [10], [31], [32]. Similarly, it has been shown that object observation consistently results in the activation of premotor and intraparietal areas, that are also active when actually using the object [12], [13], [14], [15], [16]. The present study extends these findings by showing that manipulable objects facilitate the integration of visual and tactile information in peripersonal space. That is, using manipulable objects always involves an interaction between one's body and the object and thus requires the integration of visual information about the object with multisensory information about one's own body.

The present data is in line with studies on tool use, showing that actively using a tool results in facilitated cross-modal integration of information related to the tool [4], [5], [6], [7] and with recent studies showing that grasping actions facilitate multisensory processing in peripersonal space [29], [30]. For instance, it was found that preparing a grasping action compared to a pointing action resulted in the facilitated integration of visual information presented near the target [29]. In addition to these previous findings, the present study is the first to show that the mere observation of pictures representing well-known objects facilitates cross-modal integration as well. Based on our previous experience with objects, the observation of an object likely results in the retrieval of the motor programs and body postures associated with actually using the object and thereby facilitates cross-modal integration. The close association between multisensory perception and action is in line with the notion that action and perception are mutually dependent processes [33], [34], [35]. For instance, reading words referring to actions or objects results in the activation of motor-related brain regions [36], [37]. Conversely, action preparation can facilitate the recognition of manipulable objects [38], [39] or words referring to the intended end location of the action [40]. This reciprocal relation between multisensory perception and action is likely mediated by activation in intraparietal and premotor areas, that have been implicated in retrieving conceptual knowledge supporting object use [12], [13], [14], [15], [16] and in supporting multisensory integration regarding tool use [41], [42], [43], [44].

An important question is whether tool use results in a modification of the body schema (i.e. the implicit representation of our body that guides our actions; [3], [45], [46], [47]), the body image (i.e. the explicit and conscious visual representation of our body; [48], [49], [50]) or whether tool use mainly affects multisensory processing in peripersonal space (i.e. the space directly surrounding our body; [4], [29]). First, it should be noted that the definition of the terms ‘body image’ and ‘body schema’ is a matter of ongoing debate (cf. [45], [48]) and that the more neutral term ‘body representation’ avoids the problems with demarcating the often fuzzy boundaries between the body as object of perception or action (see also: [51]). Because the CCE is enhanced when visual distractors are presented near objects that are easily integrated in the body schema, like rubber hands and handheld tools [3], [7], [28], some authors have suggested that the CCE is a measure of the integration of information in the body schema. However, other authors have suggested that the rubber hand illusion does not affect the body schema (i.e. grasping actions are not affected by the illusion) but the body image [49], [50]. In a recent study it was found that subjects experienced a feeling of ownership only for realistic prosthetic hands but not for non-corporeal objects, suggesting that these objects are not integrated [52]. Thus, rather than affecting the body schema or body image, it seems more likely that tool use mainly affects multisensory processing in peripersonal space [45] and that the stronger CCE for manipulable objects reflects a process of facilitated cross-modal integration in peripersonal space.

Our data suggests that effects of object manipulability on cross-modal integration are automatic. That is, a stronger CCE for manipulable objects was observed both when subjects were required to retrieve the action information associated with using the object (Experiment 1), but also when subjects were required to attend only to the visual properties of an object (Experiment 2). These findings are in line with earlier studies reporting similar automatic effects of object observation on the activation of motor-related information (e.g. [9], [13], [32]).

Previous studies have shown that manipulable objects facilitate the allocation of spatial attention towards the location of the graspable object [17], [18], [19], [20]. In addition, it has been found that shifting spatial attention to the relevant target location can enhance the cross-modal congruency effect [53], [54]. Accordingly, it could be that the facilitated cross-modal integration for manipulable objects actually reflects the indirect effect of the allocation of spatial attention on tactile perception (see also: [55]). This explanation would be in line with the premotor theory of attention, according to which attention is driven by a parieto-frontal network that is shared between different modalities (i.e. vision, touch and action; [56], [57]). However, it should be noted that in the present study we used only pictures representing single objects and the tactile stimuli were presented only to the right hand, thus yielding a spatial attention explanation less plausible (i.e. there was no need to attend to the left or the right side). Rather than reflecting effects of space-based attention, it could be that the stronger CCE for manipulable objects is partly driven by enhanced object-based attention [58], [59]. Future studies would need to address the effects of space- and object-based attention on cross-modal integration in more detail.

In this study all object categories were carefully matched for familiarity and visual complexity to control for the possible confound that differences in the CCE could be attributed to other factors than object manipulability. In addition, in two post-tests we controlled for the possible confound that the stronger CCE for manipulable objects could be attributed to differences in object size (i.e. manipulable objects are smaller and therefore easier to detect). The finding that the CCE was comparable between objects of medium and high manipulability provides further support for the notion that CCE was not modulated by object size, as these categories differed strongly in object size but the CCE was comparable between both categories. Thus, rather than reflecting low-level visual features, the present study suggests that cross-modal integration is primarily driven by the manipulability of the object.

The differentiation between the categories of medium and high manipulability was based on the manipulability ratings. The object category of medium manipulability represented objects that are relatively easy to manipulate (e.g. car keys, a soap dispenser, tweezers), but that are not used as frequently as objects of high manipulability (e.g. a toothbrush, a mug, a cell phone). One possible explanation for the finding that the CCE did not differ between objects of medium and high manipulability could be that the present study did not differentiate between different types of manipulability. Previous studies have shown that functional manipulability (e.g. the action required for actually using the object) should be distinguished from volumetric manipulability (e.g. the action required for picking up the object) and that both types of manipulability are associated with a differential activation in sensorimotor areas and with specific behavioral effects [60], [61], [62]. Still, the finding of a stronger CCE for objects of both medium and high manipulability, suggests that it is primarily the graspability of objects that reliably facilitates cross-modal integration rather than object familiarity. The finding that frequency of usage does not affect cross-modal integration is in line with studies on tool use, in which a relatively short training with a novel tool was already sufficient to result in a remapping of peripersonal space [4], [5], [6], [7], [8].

### Conclusions

In sum, the main finding of the present study is that the mere observation of manipulable objects facilitates the integration of cross-modal information in peripersonal space. Thereby this study extends previous findings on tools and conceptual knowledge, suggesting that one's peripersonal space can be extended or projected towards everyday objects.

## Supporting Information

Appendix S1 **BOSS Object pictures used in the experiment.**
(DOCX)
