# Supplementary material for: Manipulable Objects Facilitate Cross-Modal Integration in Peripersonal Space
Source: PLoS One. 2011 Sep 19;6(9):e24641. doi: 10.1371/journal.pone.0024641 (PMC3176228; doi:10.1371/journal.pone.0024641)
Supplement: Appendix S1 — BOSS Object pictures used in the experiment. (DOCX) [file pone.0024641.s001.docx]

alarmclock.jpg; axe.jpg; ball02.jpg; baseball01a.jpg; beermug01a.jpg; binder03b.jpg; book01b.jpg; boot02b.jpg; bottlecap.jpg; bottleopener01.jpg; bow02b.jpg; bow04.jpg; bowl01.jpg; bowl02.jpg; box01.jpg; bracelet01.jpg; brick.jpg; broom.jpg; bubbleblower.jpg; button01.jpg; candle01.jpg; candle08b.jpg; candleholder01.jpg; cardboardbox.jpg; carkeys.jpg; cellphone.jpg; chalkboard.jpg; chalkboarderaser.jpg; christmasball.jpg; clock05.jpg; comb02a.jpg; corkscrew03a.jpg; dentalfloss03b.jpg; deodorant02a.jpg; drill01b.jpg; dropper.jpg; drum.jpg; electricrazor.jpg; eraser.jpg; fan.jpg; flyswatter.jpg; folder03a.jpg; folder04.jpg; fork07b.jpg; glassmop.jpg; globe.jpg; grater01a.jpg; hairband.jpg; hairclip02.jpg; hairdryer02a.jpg; handfan01b.jpg; hanger02a.jpg; hourglass.jpg; icecubetray01a.jpg; icepack.jpg; iron01b.jpg; jewelrybox01b.jpg; kettle01.jpg; keychain.jpg; kitchenscale01a.jpg; lamp04a.jpg; lighter01.jpg; magnifyingglass01b.jpgmakeupbrush04.jpg; mallet01b.jpg; mascarabrush.jpg; mask02a.jpg ; match.jpg; measuringtape01.jpg; medal02b.jpg; monitor.jpg; nailclipper03b.jpg; nailpolish03b.jpg; notebook03a.jpg; paintbrush01.jpg; paintroller01.jpg; pencilcase.jpg; pencilsharpener02a.jpg; peppermill02b.jpg; perfume01a.jpg; pictureframe04.jpg; piggybank.jpg; pingpongracket.jpg; plate01b.jpg; plunger02.jpg; printer02.jpg; propanetank.jpg; ramekin01.jpg; razor01.jpg; rearviewmirror.jpg; remotecontrol04.jpg; ribbon04.jpg; rock01a.jpg; rollingpin01a.jpg; rug.jpg; saw02b.jpg; scale01a.jpg; screwdriver04b.jpg; seashell.jpg; sewingmachine01a.jpg; soap03a.jpg; soapdispenser01.jpg; speaker04.jpg; spraycan.jpg; squeegee01b.jpg; stapler03a.jpg; strawbasket01.jpg; studiolight.jpg; tambourine.jpg; taperecorder.jpg; thermometer02b.jpg; tile.jpg; toothpick02.jpg; tweezers02a.jpg; vase01.jpg; vase02.jpg; videocamera01a.jpg; wateringcan.jpg; wood.jpg; xylophone.jpg
